# The Roles of Host and Viral Antibody Fc Receptors in Herpes Simplex Virus (HSV) and Human Cytomegalovirus (HCMV) Infections and Immunity

**DOI:** 10.3389/fimmu.2019.02110

**Published:** 2019-09-06

**Authors:** Jennifer A. Jenks, Matthew L. Goodwin, Sallie R. Permar

**Affiliations:** ^1^Duke Human Vaccine Institute, Duke University Medical Center, Durham, NC, United States; ^2^Department of Pediatrics, Children's Health and Discovery Institute, Durham, NC, United States

**Keywords:** HSV, HCMV, herpes simplex virus, cytomegalovirus, FcR, Fc receptor, non-neutralizing antibodies, neutralizing antibodies

## Abstract

Herpesvirus infections are a leading cause of neurodevelopmental delay in newborns and end-organ disease in immunocompromised patients. One leading strategy to reduce the disease burden of herpesvirus infections such as herpes simplex virus (HSV) and human cytomegalovirus (HCMV) is to prevent primary acquisition by vaccination, yet vaccine development remains hampered by limited understanding of immune correlates of protection against infection. Traditionally, vaccine development has aimed to increase antibody titers with neutralizing function, which involves the direct binding of antibodies to viral particles. However, recent research has explored the numerous other responses that can be mediated by engagement of the antibody constant region (Fc) with Fc receptors (FcR) present on immune cells or with complement molecules. These functions include antiviral responses such as antibody-dependent cell-mediated cytotoxicity (ADCC) and antibody-dependent cellular phagocytosis (ADCP). Uniquely, herpesviruses encode FcR that can act as distractor receptors for host antiviral IgG, thus enabling viral evasion of host defenses. This review focuses on the relative roles of neutralizing and non-neutralizing functions antibodies that target herpesvirus antigens for HSV and HCMV, as well as the roles of Fc-FcR interactions for both host defenses and viral escape.

Herpesvirus infections are the leading cause of infectious brain damage in infants and a leading source of morbidity and mortality in immunosuppressed individuals. Neonatal herpes simplex virus (HSV) has 50% mortality in neonates who develop disseminated disease, even among those who receive appropriate antiviral therapy (1), and congenital human cytomegalovirus (HCMV) is the most common infectious cause of sensorineural hearing loss worldwide (2). In immunocompromised patients, HSV and HCMV infection can both cause severe end-organ disease. HSV-2 causes severe, sometimes refractory disease including orofacial and genital lesions in patients with HIV/AIDS and other immunocompromising conditions (3), and HCMV is a major infectious cause of morbidity and mortality in immunocompromised patients, such as recipients of allogeneic hematopoietic stem cell transplants (4). One strategy to reduce disease burden is to prevent primary acquisition or viral reactivation by vaccination. In fact, a vaccine to prevent HCMV has been designated a “Tier I priority” by the Institute of Medicine since 2000 (5). Yet despite major advancements in research and multiple clinical trials of HSV and HCMV vaccines over the last 20 years, development of efficacious vaccines remains elusive. These challenges may be due in part to a limited understanding of the immune correlates of protection against viral infection, as well as complex mechanisms of herpesvirus evasion of these immune responses.

Traditionally, vaccine developments for HSV and HCMV have predominantly focused on the generation of neutralizing responses to prevent primary acquisition. Neutralization occurs upon direct binding of antibodies to viral antigens by their antibody binding (Fab) regions and can often be mediated in the absence of the antibody constant (Fc) region, as in the case of isolated F(ab) or F(ab)'2 fragments which are enzyme-cleaved immunoglobulin G (IgG) that lack the Fc portions. Thus, neutralization is generally achieved by antibody masking of target cell receptor binding sites or inhibition of conformational change in viral spike proteins required for fusion between the viral lipid envelope and cellular plasma membrane (6). However, the results of animal vaccine studies and recent clinical trials of HSV and HCMV vaccines have suggested that neutralization may be only one of several antibody functions that protect against HSV and HCMV infections, respectively. A previous trial of an HSV-2 subunit vaccine targeting glycoprotein D (gD), which is required for HSV entry into cells (7), elicited robust neutralization but did not confer protection against genital HSV-2 infection (8). Similarly, a subunit vaccine against HCMV glycoprotein B (gB), which is required for viral entry (9), conferred ~50% protection in multiple phase II studies of HCMV but elicited negligible neutralizing responses against heterologous HCMV strains (10, 11).

Thus, recent vaccine efforts have aimed to measure both neutralizing and non-neutralizing antibody responses. These responses include antibody-dependent cellular cytotoxicity (ADCC), antibody-dependent cellular phagocytosis (ADCP), antibody-dependent complement deposition (ADCD), and antibody-dependent respiratory burst (ADRB), of which ADCC and ADCP occur upon engagement of Fc and Fc receptors (FcR) (Figure 1). ADCC is an adaptive immune response wherein IgG Fc-FcR engagement triggers lysis of target cells. Although ADCC activity is largely mediated by NK cells, it can also be mediated by non-NK cell populations in peripheral blood and mucosal compartments including monocytes, macrophages, and granulocytes. In ADCP, phagocytic cells such as monocytes, macrophages, neutrophils, and dendritic cells (DCs) express FcRs that enable them to efficiently uptake antibody-opsonized particles, enabling both clearance and presentation of viral antigens. The FcR most involved in the non-neutralizing antibody functions ADCC and ADCP are of the FcγR family.

**Figure 1:**
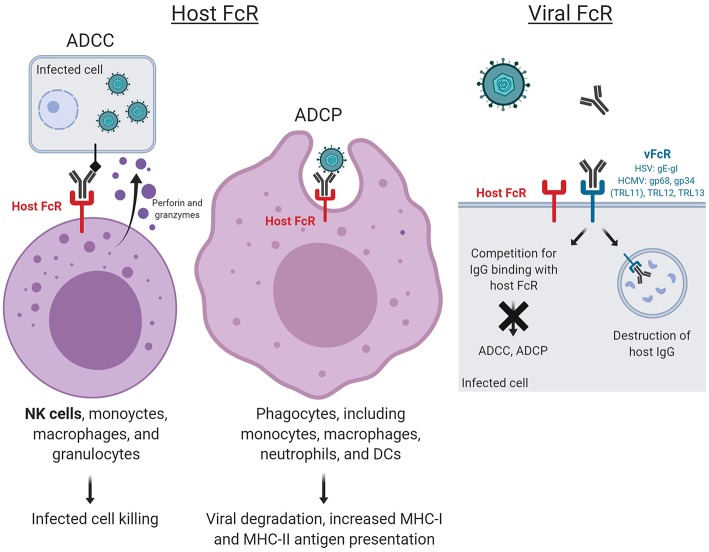
Host and virus Fc receptor (FcR)-mediated functions. ADCC and ADCP occur upon engagement of virus-specific antibody Fc fragments to FcR, resulting in cytotoxic killing of infected cells and whole virion degradation, respectively. Herpesviruses also encode their own viral FcRs (FcRs), which recognize the Fc regions of host immunoglobulins. Mimicking host FcRs, vFcRs enable herpesviruses to reduce and evade antiviral immune responses. Figure created with BioRender.

FcγR is which is one of the five main FcR classes, which includes FcγR, FcεRI, FcμR, FcαRI, and FcRn, so named for the IgG that they recognize. The FcγR family is broadly categorized into three groups: FcγRI (CD64), FcγRII (CD32), and FcγRIII (CD16), each of which coordinate different functions and are expressed on different cell types. The FcγRI family are high affinity (10^9^/M) receptors, which can bind monomeric IgG, and are constitutively expressed on monocytes and macrophages (12). By contrast, FcγRII and FcγRIII are low-affinity (10^6^/M) receptors, which bind only immune-complexed IgG, and are expressed on many hematopoietic cells. The FcγRII family is categorized into FcγRIIa, FcγRIIb, and FcγRIIc. FcγRIIa is the most widely distributed FcγR, found on neutrophils, eosinophils, B lymphocytes, platelets, mast cells, Langerhans cells, placental endothelial cells, and dendritic cells (12). In contrast to all other FcγRs which are activating, FcγRIIb is the only inhibitory FcγR, due to its unique inhibitory cytoplasmic signaling motif (13). The FcγRIII family includes two receptors: FcγRIIIa, which is expressed on monocytes, DCs, and macrophages; and FcγRIIIb, which is expressed on neutrophils, mast cells, and eosinophils. The various combinations of FcγRs play a significant role in determining an antiviral cellular response in the context of virus-specific IgG.

In humans, non-neutralizing antibody responses rely on engagement of IgG with particular FcγRs. ADCC is predominantly mediated by FcγRIIIα, FcγRI, FcγRII, FcγRIIIb, and FcαRI (CD89) (14–16). ADCP regulation is multilayered and can involve a myriad of factors, including FcR genetics, phagocyte cell type, and receptor expression pattern, tissue environment, and antibody immune complex, including specificity, isotype, subclass, and glycoforms (17). Notably, antibodies that mediate non-neutralizing functions may also mediate neutralization, and there may be not only complementary but potentially synergistic humoral effector functions for antiviral antibodies. Thus, the relative contributions of neutralizing and non-neutralizing antibody functions against HSV and HCMV for both protection and viral clearance are likely very complex.

Uniquely, herpesviruses also encode their own viral FcRs (FcRs), which recognize the Fc regions of host immunoglobulins. These vFcRs mimic host FcRs, enabling herpesviruses to reduce and evade antiviral immune responses. The elucidation of the mechanisms by which vFcRs evade host antiviral immune responses has exposed their potential as targets for novel vaccine development.

This review will discuss non-neutralizing antibody functions in HSV and HCMV, with a particular focus on functions mediated by Fc-FcR binding, as well as the role of vFcRs to mimic host FcR and to evade immune responses. An improved understanding of the distinct humoral immune correlates of protection will ultimately aid development of efficacious vaccines against herpesvirus pathogens.

## FcR-mediated Immunity Against HSV

The hallmark of HSV is its ability to establish lifelong persistent infection in sensory neurons and reactivate to cause recurrent disease or viral shedding. In HSV-infected individuals, control and clearance of the virus has been attributed to the generation of cellular immunity (18), but HSV antibodies are known to play a major role in prevention of HSV infection (19–23). In congenital HSV, maternal antibodies against HSV are known to reduce disease severity in infants (24). Women who are infected with sufficient time to transmit HSV antibodies to their infants are less likely to have infants with neonatal HSV-2 disease than women with acute HSV-2 infection at the time of childbirth (24). Thus, antibody-mediated immunity has been a central focus for HSV vaccines.

Of particular interest for HSV vaccine development are the HSV glycoproteins gD, gB, and gH/gL, which are essential for cell entry and which have been targets for multiple vaccine trials in humans (25–27). A vaccine trial of HSV-2 gD2 induced both cellular and humoral immune responses in HSV-2-seronegative patients, and despite inducing high-titer gD2-specific antibodies at levels exceeding those induced by natural infection and neutralizing antibodies, the vaccine failed to prevent HSV genital infection after 1 year of follow-up. As compared to the control group, the vaccinated demonstrated only 20% protection against genital disease (27). Surprisingly, protection against viral acquisition (with or without disease) against HSV-1 was 35% whereas there was no vaccine efficacy against HSV-2 (27). Cross-protection was expected in this trial given the high sequence homology between gD1 and gD2, yet it remained unclear what properties of the vaccine-elicited antibodies were partially protective against HSV-1 infection. In a subsequent study of the HSV-2 gD2-vaccinated women, antibody titers to HSV-2 gD2 correlated with protection against HSV-1 infection, with higher antibody concentration associated with higher efficacy, but there was no correlation between HSV-2-specific antibody titers in serum with HSV-2 protection (21). Of note, follow-up studies revealed that in sera drawn 1 month after the final dose of the HSV-2 gD2 vaccine, mean neutralizing titers to HSV-1 were 3.5 times than to HSV-2, and the mean neutralization titer against HSV-2 was 1:29, well-below that seen in natural infection (28). The results of this follow-up study may partially explain the lack of protection observed against HSV-2. Thus, although the vaccine elicited high antibody and mixed neutralizing titers to HSV but had poor efficacy against genital disease, it remains unclear if neutralization is sufficient for protection.

In addition to neutralization, recent studies have aimed to measure non-neutralizing functions of HSV-specific antibodies (Table 1). Mouse studies have revealed that passively infused intact HSV-specific IgG can protect against viral challenges by footpad injection, whereas F(ab')2 fragments, which can only mediate neutralization, confer only moderate protection, indicating the importance of Fc-mediated antibody functions against HSV (30). In mice, passive transfer of non-neutralizing monoclonal antibodies with *in vitro* ADCC activity protected complement-deficient mice against lethal HSV-2 challenge (29). Furthermore, in a murine challenge model of HSV-1 and HSV-2, a single-cycle HSV deleted of glycoprotein D (ΔgD-2), which is a major target of neutralizing antibodies, provided complete protection against lethal intravaginal or skin challenge, as well as rapid clearance and elimination of latent virus (39). Yet, interestingly, the vaccine-elicited antibodies had limited neutralization function and had enhanced FcR-mediated functions, namely ADCP and ADCC, as measured by activation of murine FcγRIII or FcγRIV, which of note is not expressed in humans but in mice is expressed on macrophages and neutrophils (39, 40, 44). Thus, both neutralizing antibodies and ADCC appear to contribute to protection against HSV in animal models.

**Table 1:** Studies implicating host FcR-mediated functions in protection against HSV and HCMV infections.

**Virus**	**Model**	**Functions implicated**	**Relevant observations**	**References**
HSV-2	Mice	ADCC	Passively transferred non-neutralizing monoclonal antibodies with known ADCC function, measured by ^51^Cr release, protected complement-deficient mice from HSV-2 challenge	(29)
HSV-1	Mice	FcR-mediated functions	Passive immunization with IgG, as compared to F(ab')2 treatment, reduced viral titer, and viral spread in HSV-1 challenged mice	(30)
HSV-2	Humans	ADCC	High maternal or neonatal anti-HSV ADCC antibody levels, measured by infected cell release of ^51^Cr label, or high neonatal antiviral neutralizing levels were independently associated with an absence of disseminated HSV infection	(31)
HSV-1	Mice	ADCC	Antibodies against HSV gB or gD given with human mononuclear cells protected against lethal challenge in neonatal mice with HSV-1, and protection was associated with monoclonal ADCC activity	(32)
HSV-1	Mice	ADCC	Both neutralization and ADCC activity were independently associated with *in vivo* protection against HSV-1 challenge	(33)
HSV-2	Humans	ADCC	Among HSV-2 gB-2 and gD-2-vaccinated subjects, low ADCC responses were implicated in poor vaccine efficacy against HSV-2	(34)
HSV-2	Mice	ADCC	Antibody dependent protection against genital HSV-2 infection occurs in an Fcγ-receptor dependent mechanism	(35)
HSV-1	Mice	ADCC	HSV-1 FcγR protected the virus by blocking IgG Fc-mediated complement activation and NK cell-mediated ADCC *in vivo*.	(36)
HSV-2	Mice and guinea pigs	Not specified	Neutralization and IFNγ T cell responses did not correlate with vaccine efficacy for HSV-2 subunit vaccines containing gD or gB alone or in combination, together with CpG adjuvant	(37)
HSV-2	Mice	ADCC	The majority of sera collected from mice immunized with mature gG-2 plus CpG adjuvant showed complement-mediated cytolysis and macrophage-mediated ADCC, measured by infected cell release of ^51^Cr label, but not neutralization	(38)
HSV-1 and HSV-2	Mice	ADCC	Single-cycle HSV ΔgD-2 vaccine conferred protection against skin challenge with clinical isolates, as well as rapid clearance and elimination of latent virus. Protection was associated with target cell killing	(39)
HSV-1 and HSV-2	Mice	ADCC, ADCP	Single-cycle HSV ΔgD-2 vaccine conferred protection against skin challenge with clinical isolates, and protection was associated with activation of HSV-specific murine FcγRIII and FcγRIV	(40)
HSV-1	Human mAbs	ADCC	mAbs derived from humans vaccinated with the HVEM binding domain of HSV-1 gD mediated neutralization and ADCC, measured by NK cell activation, and reduced ocular disease in infected mice	(41)
HSV-1 and HSV-2	Mice	ADCC, ADCP	Single-cycle HSV ΔgD-2 vaccine conferred protection against skin challenge with clinical isolates, and protection was associated with activation of HSV-specific murine FcγRIV	(42)
HCMV	Mice	Not specified	Prophylactic treatment with HCMV gB-specific neutralizing and non-neutralizing antibodies protected equally against CMV challenge. In the setting of established infection, neutralizing and non-neutralizing antibodies provided protection, with neutralizing antibodies being superior	(43)
HCMV	Humans	ADCP	An HCMV gB vaccine that afforded 50% protection in a clinical trial in post-partum women elicited limited neutralization of autologous virus and negligible neutralization of heterologous strains but robust ADCP	(10)
HCMV	Humans	ADCP	An HCMV gB vaccine that afforded partial protection in a clinical trial in transplant recipients elicited limited neutralization of autologous virus and negligible neutralization of heterologous strains but robust ADCP	(11)

*gB, glycoprotein B; gD, glycoprotein D; IFNγ, interferon-gamma; gG, glycoprotein G; HSV ΔgD-2, HSV deleted of glycoprotein D*.

In human studies, non-neutralizing antibody functions are correlated with protection against infection. In follow-up studies of the HSV-2 gB2 and gD2 combination vaccine, which failed to confer protection against HSV-2 in HSV-2-seronegative women, found that the vaccine induced neutralization but had limited ADCC, as measured by target cells activation (34). A neonatal herpes study evaluated both neutralizing antibodies and ADCC titers in newborns and noted that each independently correlated with protection against neonatal HSV infection (31). These results were also recapitulated in mice (32). Previous vaccine studies also trialed a recombinant HIV glycoprotein 120 (gp120) construct fused to the HSV-1 gD herpesvirus entry mediator binding domain (HVEM) (41), which is a cellular receptor for HSV and is expressed on lymphocytes, fibroblasts, and epithelial cells (45). Monoclonal antibodies isolated from HVEM-vaccinated individuals had both neutralization and ADCC function (45). In an *in vivo* challenge model, these human monoclonal antibodies from HVEM-vaccinated subjects protected mice from lethal infection and resulted in reduced disease burden, namely reduced ocular disease and modestly reduced virus shedding and latency after corneal inoculation with HSV-1 (45). These studies indicate the importance of Fc-mediated functions, namely ADCC, in protection against HSV in both humans and murine models and are under current investigation in HSV vaccine development.

Immunoglobulin G (IgG) genetic variations and FcγR polymorphisms are known to exert effects on ADCC functions, although this has not yet been explored extensively in the context of HSV. Previous studies have demonstrated that homozygosity for the higher-affinity allele CD16A-158V (which encodes FcγR3α) protects against symptomatic HSV-1 infection, whereas the CD32A-131H/R (which encodes FcγR2α-C) dimorphism does not (46). In a follow-up study, NK cell degranulation was consistently enhanced against opsonized HSV-1-infected targets in specifically CD16A-158V/V carriers as compared with CD16A-158F/F carriers (47). Other genetic polymorphisms for IgG and FcγR in the context of non-neutralizing antibody functions such as ADCC warrant future study.

## FcR-mediated Immunity Against HCMV

Many current vaccine strategies against HCMV infection have been designed to induce neutralizing antibody responses (48–53). However, it remains unclear whether HCMV transmission will be impacted by plasma neutralization, as reinfection occurs routinely in individuals with pre-existing immunity. *In vivo* HCMV is known to be largely cell-associated, spreading intracellularly and via cell-to-cell without diffusing into extracellular spaces as a cell-free virion (54), and clinical strains *in vitro* recapitulate this feature (54, 55). Yet, *in vitro* studies of HCMV have largely relied on laboratory strains that produce high titers of cell-free virus (56), which may be more vulnerable to neutralizing antibodies, IFN, and cellular restriction factors, as compared with virus transmitted by cell-free entry. A reconstructed wild-type HCMV strain that spread via direct cell-cell contact demonstrated that high expression of the pentameric gH/gL/gpUL128-131A complex enabled resistance to neutralizing antibodies, providing insight into potential mechanisms that facilitate the *in vivo* persistence of HCMV (57).

Although early studies had suggested that neutralizing antibodies may be protective against congenital HCMV transmission, recent randomized controlled trials in humans have indicated that neutralizing antibodies are insufficient to protect against congenital transmission, implicating a potentially important role for FcR-mediated non-neutralizing antibody responses. In a 2005, non-controlled study of HCMV congenital transmission, administration of HCMV-specific hyperimmune globulin to pregnant women with primary infection decreased the rate of mother-to-fetus transmission from 40 to 16% (*p* = 0.04), and the risk of congenital disease decreased from 50 to 3% (*p* < 0.001) (58). Subsequent non-randomized studies showed a decrease in the number of congenitally infected infants born to mothers who had been treated with hyperimmune globulin or improved outcomes in HCMV-infected infants (59–62). However, in a randomized clinical trial, the administration of polyclonal human IgG containing high titers of neutralizing antibodies failed to prevent congenital infection (63). Regarding primary infection, the most efficacious HCMV vaccine to-date was a protein subunit vaccine targeting HCMV glycoprotein B (gB), which is essential for viral entry into all cell types (9), with an MF59 adjuvant (gB/MF59), and although it achieved 50% protection against primary acquisition in multiple phase two clinical trials (64–66), sera from gB/MF59 vaccinees exhibited poor neutralization of heterologous HCMV strains (10, 11). Furthermore, a correlation between anti-gB antibody titers and protection in vaccinated transplant recipients was found to be independent of neutralization activity (11). These results suggested that the partial protection conferred by the gB/MF59 vaccine was not due to neutralizing antibodies but perhaps due to non-neutralizing antibody responses.

Follow-up studies have aimed to better characterize FcR-mediated non-neutralizing responses protective against HCMV (Table 1). Although the HCMV gB/MF59 vaccine did not elicit neutralizing antibodies against heterologous HCMV strains in populations of post-partum women and transplant recipients, sera from post-partum vaccinees mediated robust ADCP of both gB protein-coated beads and fluorescently-labeled whole HCMV virions by human monocytes (10, 11). Interestingly, the gB/MF59 vaccine preferentially induced high binding magnitude gB-specific responses of the IgG3 isotype (10), which is known to demonstrate high avidity for FcR on monocytes and macrophages and which has been shown to coordinated multiple antibody effector functions including ADCC and ADCP (67, 68). Vaccine-elicited antibody enhancement of phagocytosis is thought to have contributed to the partial efficacy of the HCMV gB subunit vaccine, though it remains unclear if ADCP is necessary or sufficient for protection against disease and warrants further study.

In HCMV, ADCC appears to play a role in antiviral immunity for naturally infected individuals, but its importance in protection for vaccine-elicited responses remains to be determined. Studies of pooled human IgG from naturally seropositive individuals (Cytogam) can promote antibody-mediated NK cell lysis (69), and ADCC is measurable in naturally seropositive subjects (10). However, postnatal and transplant subjects vaccinated with gB/MF59 demonstrated no substantial ADCC-promoting antibody response in *in vitro* assays with human NK cells (10, 11). In a murine model of CMV infection, prophylactic administration of HCMV gB-specific monoclonal antibodies before infection was also protective, and both neutralizing and non-neutralizing mAbs were equally effective in preventing lethal infection of immunodeficient mice (43). Thus, FcR-mediated non-neutralizing antibody functions such as ADCP and ADCC against HCMV appear to be involved in the antiviral immune response, but their separate and overlapping contributions with neutralizing responses remain to be determined.

## HSV and HCMV Viral FcR in Immune Evasion

Uniquely, members of the α- and β-subfamily of *herpesviridae* establish permanent, lifelong infections in their hosts. They achieve this in part by encoding surface glycoproteins that bind to the Fc region of host IgG and facilitate evasion from the host immune response (70). HSV and HCMV encode a number of immunomodulating proteins such as decoy receptors and chemokines, which are theorized to protect against both innate and adaptive immune responses (71).

HSV-1 encodes surface glycoproteins gE and gI, which can form a complex on infected cells or on the virion surface that binds to the Fc domain of host IgG (72, 73). This complex acts as a vFcR and is associated with cell-to-cell spread of infection (72, 73). The HSV gE-gI complex is required for the binding of monomeric non-immune IgG, but HSV gE alone is sufficient for binding polymeric IgG (74). The HSV gE-gI complex is thought to facilitate degradation of antiviral host antibodies through pH-specific binding. In this process, host anti-HSV IgG antibodies participate in antibody bipolar bridging, whereby an HSV-specific host antibody simultaneously binds to the HSV gE-gI complex with its Fc region and to a specific HSV-antigen (e.g., gC or gD) with its Fab arms (75–78). At the basic pH of the cell surface, anti-HSV antibody can bind to both HSV gE-gI complex and HSV antigen, but once this antibody is endocytosed and trafficked into the late endosomes, the HSV gE-gI complex dissociates from the antibody Fc region. The host antibody bound to HSV antigen is then localized to the lysosome, where both are degraded, whereas the HSV gE-gI complex can be recycled back to the cell surface. This process of antibody bipolar bridging protects virally infected cells from antibody- and complement-dependent neutralization (78), ADCC (36), and granulocyte attachment (79), and is thus an important mechanism of host immune evasion from antibody-mediated clearance.

One novel strategy for vaccine development against HSV infection aims to prevent these viral immune evasion activities (Figure 1). In fact, a trivalent HSV vaccine composed of the vFcγR HSV-2 glycoproteins C, D, and E has been tested in animal challenge studies, in which the vaccine protected seronegative rhesus macaques against intravaginal challenge and seronegative guinea pigs against severe genital disease (80). These glycoproteins were selected due to the involvement of HSV-2 gC in complement cascade inhibition, thus contributing to immune evasion (81); gD in virus entry (26); and gE in blocking host IgG Fc thus also contributing to immune evasion (82). Immunogenicity data revealed that the vaccine induced plasma and mucosa neutralizing antibodies, antibodies that block gC2 and gE2 immune evasion activities, and stimulated CD4 T cell responses (80). In guinea pigs previously infected intravaginally with HSV-2, the vaccine reduced the frequency of recurrent genital lesions and the frequency and duration of vaginal shedding. These studies demonstrate the potential for vaccine candidates aimed at preventing HSV evasion from host defenses in the context of both primary infection and reactivation and require further studies in humans.

Human HCMV encodes four glycoproteins that act as vFcγR and interfere with IgG-mediated immunity against HCMV: gp68, gp34 (toll-like receptor 11/TLR11), TLR12, and TLR13 (83–85), each with a unique binding pattern to host IgG. Distinct from host FcγR, HCMV vFcγR demonstrate glycan independent binding (86), and all HCMV FcyR genes are transcribed with relatively delayed kinetics during the protracted viral replication cycle, reaching abundant protein amounts during the late phase of infection (83). HCMV gp68 and gp34 are specific for binding human IgG but do not discriminate among the IgG subclasses (87). Recent studies reported formation of antibody bipolar bridging complexes with gp68 and with gp34, and that HCMV lacking gp34 or/and gp68 elicited much stronger activation of host FcγRI, FcγRIIA, and FcγRIIIA by polyclonal HCMV-immune IgG as compared to wildtype HCMV (71). These results implicate HCMV gp34 and gp68 in evading the host FcR-mediated immune response. Unlike the HSV-1 gE-gI complexes, the gp68-Fc interaction is broadly stable across acidic and basic pHs (86), resulting in degradation of the HCMV vFcγR gp68 with the host antibody and HCMV antigen. It is clear that vFcRs are a unique viral immune evasion factor, and further investigation will be required to understand the role of these receptors in both viral pathogeneses, and as potential novel targets for vaccine development.

## Conclusion

Herpes simplex virus (HSV) and HCMV infections are a serious cause of morbidity and mortality among infants and immunocompromised patients worldwide. There is an urgent need for efficacious vaccines against these pathogens, both to prevent primary acquisition as well as reactivation of latent virus. Historically, vaccine development has aimed to increase the titer of neutralizing antibodies against HSV or HCMV to confer protection, but recent clinical trial data and follow-up immunogenicity studies have investigated the roles of antibody Fc-mediated functions, namely ADCC and ADCP. Furthermore, herpesviruses uniquely encode vFcRs that promote destruction of antiviral host IgG and may enable immune evasion. An improved understanding of non-neutralizing antiviral immune responses and herpesvirus vFcRs may illuminate new pathways for the development of more efficacious vaccines against HSV and HCMV infections.

## Author Contributions

JJ wrote the majority of the manuscript. MG wrote and edited the manuscript. SP is the PI of JJ and MG. She oversaw the writing and made significant editing contributions.

### Conflict of Interest Statement

The authors declare that the research was conducted in the absence of any commercial or financial relationships that could be construed as a potential conflict of interest.
